# Single nucleotide variations in the *ANXA5* promoter regulated piglet weight in the Min pig

**DOI:** 10.3389/fvets.2025.1644944

**Published:** 2025-10-20

**Authors:** Mingyang Han, Yingkun Zhang, Yu Zhang, Diwen Yao, Shupin Sun, Xiaoji Li, Xiuqin Yang, Shengwei Di, Xibiao Wang, Jiancheng Cai, Liping Chen, Lihe Dai, Buyue Niu

**Affiliations:** ^1^College of Animal Science and Technology, Northeast Agricultural University, Harbin, China; ^2^Lanxi Breeding Farm, Lanxi, China; ^3^Zhejiang Mebolo Swine Breeding Co., Ltd., Jinhua, China; ^4^Zhejiang Key Laboratory of Livestock and Poultry Biotech Breeding, Institute of Animal Husbandry and Veterinary Science, Zhejiang Academy of Agricultural Sciences, Hangzhou, China

**Keywords:** *ANXA5*, *NR4A1*, functional SNP, production trait, Min pigs

## Abstract

*ANXA5* is a pleiotropic candidate gene, however its effect on piglet production trait remains unclear. In this study, a novel SNP: g.-519 A > G was identified in ANXA5 promoter. In Min pigs, association analysis showed the birth weight of AA animals at g.-674 C > A was higher than that of the AC or CC piglets (*p* < 0.05), while the heterozygous of g.-519 A > G had a higher weight than the homozygote at day 3, 7, 14, 21 after birth (*p* < 0.05). Porcine *ANXA5* and *NR4A1* existed in piglets’ gastrointestinal tract, and NR4A1 localized to the nucleus and cytoplasm which regulated the expression of *ANXA5*. Luciferase reporter analysis demonstrated that the deletion of predictive NR4A1 binding region decreased the luciferase activity of porcine *ANXA5* promoter (*p* < 0.05), and the A allele of g.-519 A > G within this region had significantly higher luciferase activity than the G allele (*p* < 0.01). In conclusion, this research suggested that g.-519 A > G was a piglet weight variant that regulated the transcription of *ANXA5* partially by NR4A1.

## 1 Introduction

The genetic improvement of reproduction trait is important for the porcine production. In the past decades, gene-based selection for female fertility has increased the ovulation rate and the number of piglets born (1). However, the proportion of low birth weight piglets increased accordingly (2–4). Additionally, selection for larger litter size produced sows with a repeatable low average litter birth weight phenotype (3). Piglets’ low birth weight is associated with increased mortality, high infection risk, poor growth and meat quality (5, 6). Hence, it is encouraged to identify pleiotropic genes or genetic variations, which contributes to understand the genetic mechanism of complex traits and benefits porcine production.

As an important member of Annexin protein family, AnnexinA5 (*ANXA5*) is linked to multiple functions, such as placental anticoagulant, cell apoptosis, immune response, and so on (7, 8). Häggman and Uimari (9) located a putative lethal haplotype on SSC8 in Yorkshire boars, confirmed the effect of this haplotype on the number of stillborn piglets, and suggested *ANXA5* as the positional and functional candidate gene for swine reproduction and fertility traits. Intra-uterine growth restriction (IUGR) is a critical factor for piglets’ mortality, which impairs individual development and reduces the birth weight. Wang et al. (10) found *ANXA5* expressed differentially in the jejunal mucosa of IUGR and normal piglets. In order to understand the genetic interplay between litter size and production traits in the Yorkshire, Wei et al. (11) reported a set of pleiotropic candidate genes by integrative genomic strategy. Among them, *ANXA5* was associated with total number of piglets born (TNB), number of piglets born alive (NBA) and mean litter weight (MLW). In human, it is clear that the H2 haplotype on *ANXA5* promoter increases the risks of placental thrombus and pregnancy loss through reducing the expression of *ANXA5* (12–14). Consistently, our recent study confirmed the existence of functional genetic variation associated with semen traits in porcine *ANXA5* promoter (15). However, the effect of these variations on the piglets’ production trait is still unknown.

The Min pig is the most famous indigenous breed in Northeast China. Compared to the global general breeds, Min pigs have superior meat quality, high reproductive capacity, and strong stress resistance^1^. However, the slow growth rate of Min pigs limits their utilization in commercial production. In terms of the multifunction of *ANXA5*, the purpose of this study was to evaluate the effect of *ANXA5* on the growth of Min piglets, explore the biological function of the identified mutations, thereby providing new insights into the pleiotropic genetic architecture, the conservation and utilization of Min pigs.

## 2 Materials and methods

### 2.1 Animals

Tissue samples (stomach, duodenum, jejunum, ileum, cecum, colon, and rectum) from 4 piglets were described by Niu et al. (16). A total of 186 Min piglets were raised in the Lanxi Breeding Farm (Lanxi, Heilongjiang, China). All these piglets were weighed (at birth, day 3, 7, 14, and 21) and weaned at day 35. Average daily gain (ADG) was calculated using the formula: ADG = (21-day weight - birth weight) /21. 84 Jinhua pigs were raised in the Zhejiang Mebolo Swine Breeding farm, and 16 Durocs were provided by Haining Yangdu Science and Technology Ranch of Zhejiang Academy of Agricultural Sciences, China.

### 2.2 Genotyping SNPs within the promoter of porcine *ANXA5*

All the primers were designed based on porcine *ANXA5* (GenBank accession: XM_003129218.5) and *NR4A1* (Ensembl: ENSSSCT00000059252.3) by Primer 5 (Table 1). For the PCR, the 20 μL reactions contained 100–150 ng DNA, 10 μL 2 × Taq Master Mix (Takara, Dalian, China), and 0.5 μM primers (Table 1), performed under the following conditions: 94 °C for 4 min; 35 cycles of 94 °C/30 s, primer-specific annealing (Table 1)/30 s, 72 °C/1 min; final extension at 72 °C/10 min. For the *Hha* I PCR-RFLP, the DNA fragments containing SNP: g.-674 C > A were amplified using primer pair ANXA5-HhaI (Table 1) from 186 Min pigs. Then, the 8.5 μL of PCR-amplified products, 1 μL of 10 × M Buffer, and 0.5 μL of *Hha* I enzyme (Takara, Dalian, China) were mixed, incubated at 37 °C for 8 h and detected by 1.5% agarose gels electrophoresis.

**Table 1:** Primers used for real-time PCR, SNPs identification and plasmids construction.

Primer	Primer sequence (5′-3′)	Annealing temp (°C)	Product size (bp)	Binding region
NR4A1	F: TTAGAATTCGCATGCCCTGTATCCAAGCCCAA	60	1797	-
	R: ATTGGTACCTCAGAAGGGCAGCGTGTCCATAAAG			
EGFP	F: TTAGAATTCGCATGGTGAGCAAGGGCGAGGA	60	726	-
	R: ACAGGGCATCTTGTACAGCTCGTCCAT			
ANXA5-J5	F: TATGGTACCCGGAAGCGTGTCCCTACT	58	818	−493
ANXA5-R (15)	R: CCGCTCGAGGCGATTTTCTGGATTTTGG	-	-	-
ANXA5-P-GG	F: CTGCGCAA**G**GGCCAGGCGGT	-	-	-
	R: ACCGCCTGGCC**C**TTGCGCAG			
NR4A1-Exp	F: TGGACAAGAGGCGGCGAAAC	55	177	-
	R: GGACCAGGGAGGTGAGGAGATT			
GAPDH-Exp	F: CCCCAACGTGTCGGTTGT	55	83	-
	R: CCTGCTTCACCACCTTCTTGA			
ANXA5-SSCP	F: GAGGTCACGGAGGGGAGT	59	124	-
	R: TGGAACTCAGTAGGGACACG			
ANXA5-HhaI	F: TTGAAAGTTCTAGGCTGGTT	52	327	-
	R: ACTCTAGGTTTCGGGTGC			

The underline shows the additional restriction sites: *Kpn I* (GGTACC), *Xho I* (CTCGAG), and *EcoR I* (GAATTC). The bolded bases are the mutated bases.

The sequence of *ANXA5* promoter from Min pigs and Landraces obtained in our previous study (15) was re-aligned by DNAMAN^2^. The putative functional transcription factor binding sites were analyzed by JASPAR^3^. For the SNP: g.-519 A > G, the DNA fragments containing this SNP were amplified using primer pair ANXA5-SSCP in 186 Min pigs (Table 1). Then, the 1 μL PCR product was mixed with 9 μL denaturing buffer and denatured at 98 °C for 10 min. After this, the mixture was immediately cooled in ice water for 5 min, separated by 14% polyacrylamide gel electrophoresis (PAGE), and finally analyzed by silver staining. Alternatively, the PCR products containing this SNP were amplified by primers: ANXA5-S-F/R (15), purified and sequenced commercially (Sangon, Shanghai, China).

### 2.3 Plasmids construction

The pGL3-ANXA5-J3 containing the putative NR4A1 and ESR1 binding sites and the pGL3-ANXA5-P containing A allele at g.-519 A > G of the *ANXA5* promoter were constructed in our previous study (15). In this study, with the pGL3-ANXA5-J3 as template, the 5’ NR4A1 binding region deletion fragments were produced using primers: ANXA5-J5-F/R (Table 1) and named ANXA5-J5. With pGL3-ANXA5-P as template, a mutant fragment with G allele which caused the loss of the putative NR4A1 binding region was amplified using mutagenic primer: ANXA5-P-GG-F/R (Table 1) and termed ANXA5-GG. Then, the fragment of ANXA5-GG was digested by *Kpn* I and *Xho* I (Takara, Dalian, China), inserted into pGL3-Basic vector (Promega, Madison, WI, USA), confirmed by DNA sequence and double restriction endonuclease digestion, which was named pGL3-ANXA5-GG, respectively.

The complete coding sequence of porcine *NR4A1* was amplified using primers NR4A1-CDS-F/R (Table 1), digested and cloned into the *Kpn* I/*EcoR* I restriction sites of the pCMV-HA expression vector, confirmed and named pCMV-HA-NR4A1.

The coding sequence of both *EGFP* and porcine *NR4A1* were amplified using primer pairs EGFP-F/R and NR4A1-F/R, respectively (Table 1). With the mixture of these two fragments as template, overlap extension PCR was performed with primers EGFP-F and NR4A1-R to generate the NR4A1-EGFP fusion fragment. After purification and sequencing, the NR4A1-EGFP fragment was inserted into the *EcoR* I/ *Kpn* I sites of pCMV-HA to construct the pCMV-HA-EGFP-NR4A1.

### 2.4 Cell transfection and luciferase reporter assay

The ST and IPEC-J2 cells were cultured with DMEM supplemented with 10% FBS (Gibco, Carlsbad, CA, USA) and 1% penicillin–streptomycin solution (Beyotime, Shanghai, China), maintained at 37 °C in the atmosphere of 5% CO_2_. For the promoter activity assay, cells were seeded in 24-well plates. To explore the role of the putative NR4A1 binding site within the *ANXA5* promoter, with 1.5 μL of LP2000 Transfection Reagent (Invitrogen, Carlsbad, CA, USA), the cells were transiently transfected with 0.5 μg of the corresponding luciferase reporter gene vectors (pGL3- ANXA5-J3, pGL3- ANXA5-J4 and pGL3- ANXA5-J5), or 0.005 μg of pGL3-basic as negative control and pRL-TK as internal control. To validate the role of NR4A1 on the transcription of *ANXA5*, the cells were co-transfected with 0.25 μg of the pCMV-HA-NR4A1 or pCMV-HA, 0.25 μg of the pGL3-ANXA5-P(AA) or pGL3- ANXA5-GG, and 0.005 μg of pRL-TK, with 1.5 μL of LP2000 (Invitrogen, Carlsbad, CA, USA). 24 h after transfection, in line with the Dual Luciferase Reporter Assay System (Beyotime, Shanghai, China), all the cells were harvested and lysed, and the enzymatic activity of firefly and Renilla luciferase were assessed using the Sirius L Luminometer (Berthold, Pforzheim, Germany). Relative luciferase activity was calculated as the ratio of firefly to Renilla luciferase activity (Fluc/Rluc). All transfections were performed in triplicate and repeated three times.

### 2.5 The subcellular distribution of *NR4A1*

The localization of porcine *NR4A1* in the cells was firstly predicted by PSORT II^4^ and Uniprot^5^. Then, the pCMV-HA-EGFP-NR4A1 or pCMV-HA-EGFP were transfected into the cells, respectively. After 48 h, 1 mL of 1 × Hoechst 33342 was added into each well, incubated at room temperature in the dark for 10 min. Then, the staining solution were discarded, all the cells were washed with PBS for three times. The subcellular distribution of NR4A1-EGFP was observed using the Leica TCS SP8 confocal microscope (Leica Microsystems, Wetzlar, Germany). The experiment was repeated three times with consistent results.

### 2.6 Quantitative RT-PCR (qRT-PCR)

Total RNA was extracted from specific tissues of four Min piglets, ST and IPEC-J2 cells (*n* = 3 biological replicates) using TRIzol (Takara, Dalian, China), reverse-transcribed into cDNA by PrimeScript RT Master Mix (Takara, Dalian, China). According to the manufacturer’s instructions of the TB Green Premix Ex Taq II (Tli RNaseH Plus) kit (Takara, Dalian, China), the qRT-PCR reactions (20 μL) contained 100 ng cDNA, 10 μL SYBR mix (Takara, Dalian, China), and 0.2 μM primers (Table 1), performed on the ABI QuantStudio 3 system (Applied Biosystems, Foster City, CA, USA) with: 95 °C/30s; 40 cycles of 95 °C/5 s and 60–62 °C/35 s; melt curve analysis (60–95 °C, 0.3 °C/s). The qRT-PCR for each sample was performed in technical triplicate. GAPDH-normalized mRNA levels were quantified by the 2^−ΔΔCT^ method.

### 2.7 Statistical analysis

Statistical analyses were performed using SPSS (IBM, Armonk, NY, USA). The mRNA expression of *ANXA5* or *NR4A1* in different tissues were analyzed by ANOVA (Analysis of Variance). The differences of gene expression between the cells, or the transcriptional activity between groups were evaluated using the two-tailed Student’s t-test. All data are presented as the mean ± standard error of the mean.

The effect of SNP on growth traits of 186 piglets was evaluated by general linear model (GLM) of SAS version 9.2.21 (SAS, Cary, NC, USA) as follow:

Y_ij_ = *μ* + G_i_ + S_j_ + e_ij_, where Y_ij_ is the observed traits under study, μ is the mean value of the trait, G_i_ is the genotype effects, S_j_ is the maternal effects and e_ij_ is the random residual. Significance was declared at *p* < 0.05 or *p* < 0.01.

## 3 Results

### 3.1 g.-674 C > A in the promoter of *ANXA5* was associated with piglets’ weight in Min pigs

**Figure 1:**
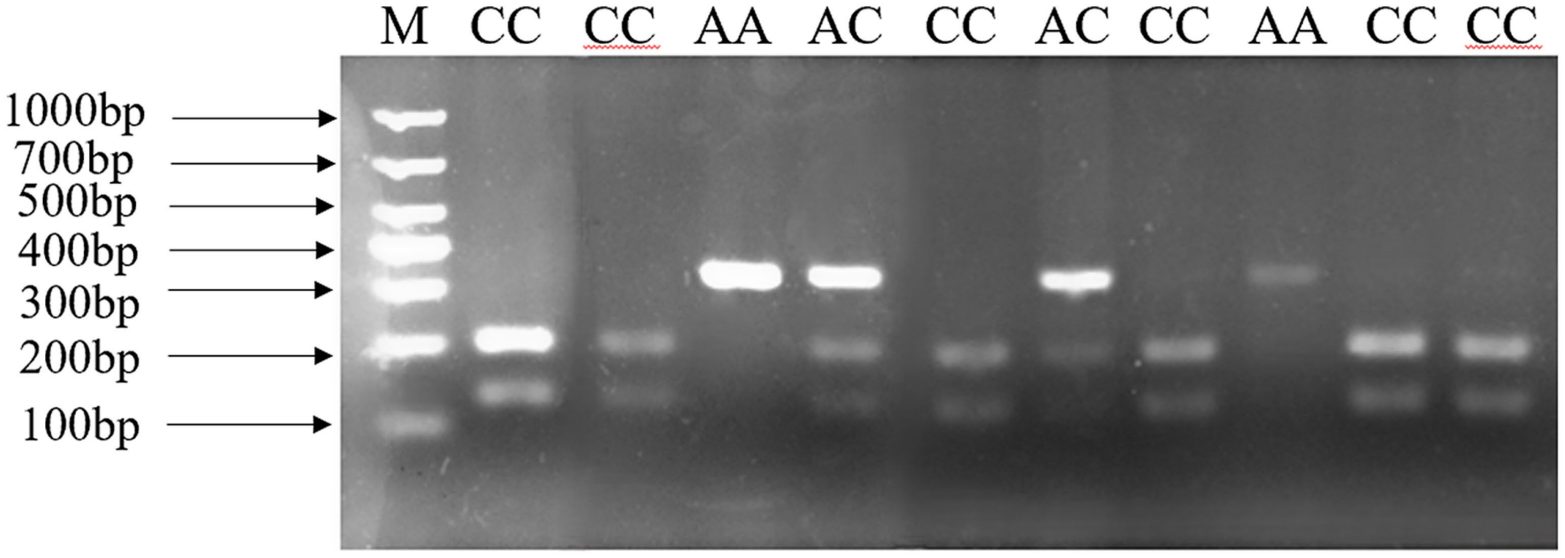
PCR-RFLP assay for g.-674 C > A in the promoter of porcine *ANXA5.*

In our previous research (15), three tightly linked SNPs which constructed one haplotype were found in the *ANXA5* promoter. In this study, a *Hha* I PCR-RFLP assay based on SNP: g.-674C > A was established to genotype this haplotype. As shown in Figure 1, a 327 bp fragment was produced by PCR and three genotypes including AA (327 bp), AC (327, 198, 129 bp) and CC (198, 129 bp) were distinguished after the *Hha* I digestion and the agarose gels electrophoresis. Association analysis in Min pigs showed that the birth weight of the AA animals was significantly higher than that of the AC and CC genotypes (*p* < 0.05) (Table 2).

**Table 2:** Association analysis between g.-674C > A and growth trait in Min pigs.

Traits	Genotypes		
	AA	AC	CC
Number	30	74	44
Birth weight (kg)	1.15 ± 0.04^a^	1.05 ± 0.03^b^	1.04 ± 0.03^b^
3-day body weight (kg)	1.32 ± 0.05	1.26 ± 0.03	1.23 ± 0.04
7-day body weight (kg)	1.78 ± 0.08	1.77 ± 0.05	1.72 ± 0.06
14-day body weight (kg)	2.37 ± 0.13	2.36 ± 0.08	2.32 ± 0.10
21-day body weight (kg)	3.47 ± 0.19	3.35 ± 0.12	3.35 ± 0.15
ADG (kg/d)	0.11 ± 0.01	0.11 ± 0.00	0.12 ± 0.01

Different lowercase letters indicate significant difference (*p* < 0.05) and capital letters indicate significant difference (*p* < 0.01).

### 3.2 g.-519 A > G in the promoter of *ANXA5* was associated with piglets’ weight in Min pigs

**Figure 2:**
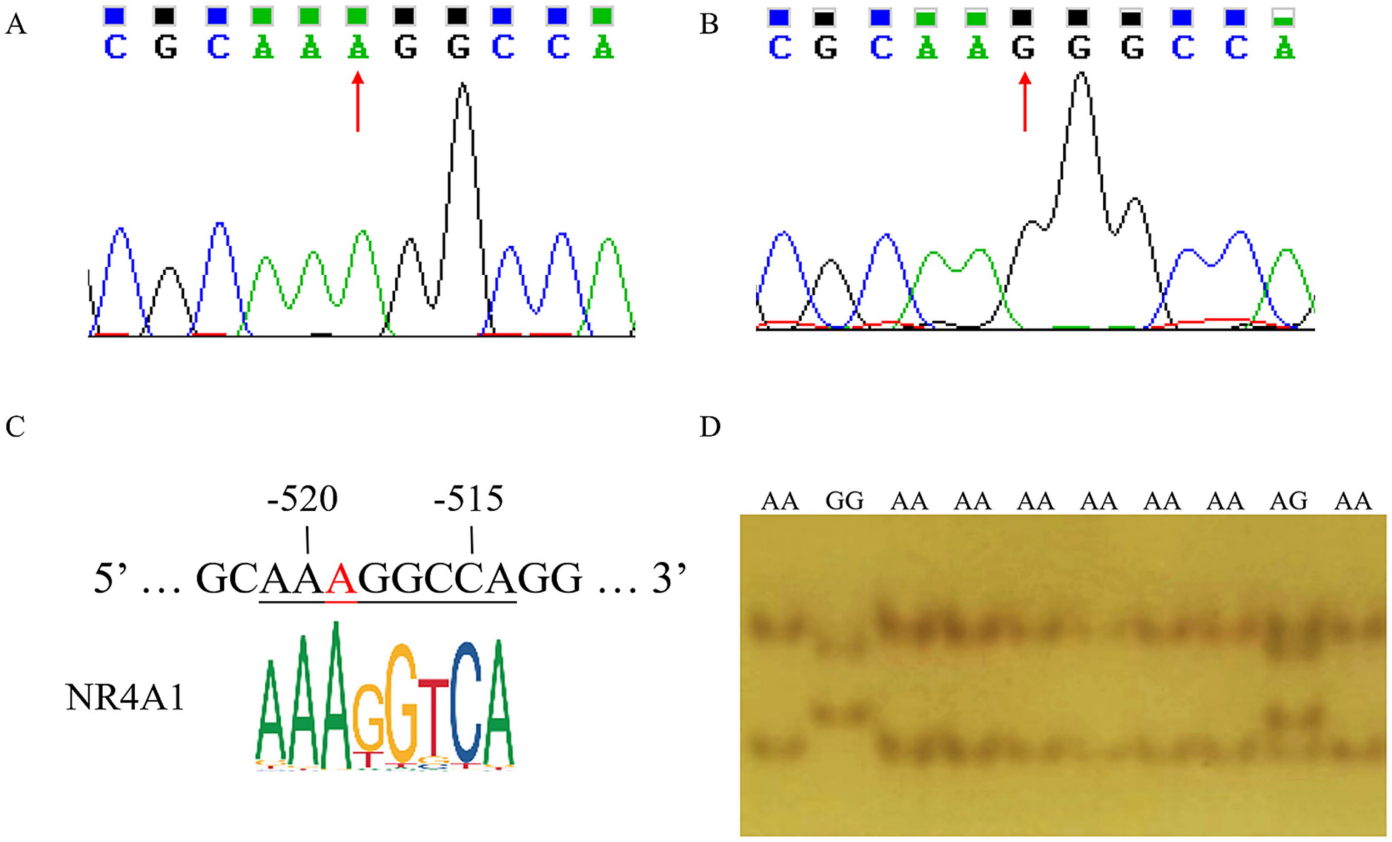
Identification of g.-519 A > G in the promoter of porcine *ANXA5*. **(A)** Sequencing result of A allele. **(B)** Sequencing result of G allele. **(C)** The transcription factor NR4A1 binding site. **(D)** PCR-SSCP assay for g.-519 A > G in the promoter of porcine *ANXA5.*

By sequence alignment, a novel g.-519 A > G located in the NR4A1 binding site (−523 bp to −512 bp before the ATG) of the *ANXA5* promoter was identified in Min pigs instead of Landraces or Yorkshires (Figures 2A,B). As predicted by JASPAR, transcription factor NR4A1 would bind to the *ANXA5* promoter with A allele but not the G allele (Figure 2C). Then, a PCR-SSCP assay was established to genotype this SNP in Min pigs, where the 124-bp fragment was obtained using primer pairs ANXA5-SSCP-F/R (Table 1) and three genotypes (AA, AG and GG) were observed through polyacrylamide gel electrophoresis (Figure 2D). This novel SNP was genotyped in Min pigs, Jinhua pigs and Durocs. As shown in Table 3, the A allele frequency was 0.50 in Min pigs, while it was 0.85 in Jinhua pigs and fixed in Durocs. Statistical analysis in Min pigs showed that AG animals had higher weight at day 3 and day 7 when compared with GG (*p* < 0.01) or AA piglets (*p* < 0.05 or *p* < 0.05), and the weight of AG individuals at day 14 or 21 were higher than that of AA piglets (*p* < 0.05) (Table 4).

**Table 3:** Genotype and allele frequencies of SNP g.-519 A > G in different pig breeds.

SNP locus	Breeds	Number	Genotype frequency			Allelic frequency	
			AA	AG	GG	A	G
SNP g.-519 A > G (rs81402897)	Min pig	186	0.435 (81)	0.135 (25)	0.430 (80)	0.502	0.498
	Jinhua pig	84	0.702 (59)	0.298 (25)	0	0.851	0.149
	Duroc	16	1 (16)	0	0	1	0

**Table 4:** Association analysis between SNP g.-519 A > G and growth traits in Min pigs.

Traits	Genotypes		
	AA	AG	GG
Number	81	25	80
Birth weight (kg)	1.04 ± 0.02	1.10 ± 0.04	1.01 ± 0.02
3-day body weight (kg)	1.22 ± 0.03^b^	1.37 ± 0.05^A, a^	1.20 ± 0.03^B^
7-day body weight (kg)	1.70 ± 0.05^B^	1.95 ± 0.08^A^	1.68 ± 0.05^B^
14-day body weight (kg)	2.20 ± 0.07^b^	2.57 ± 0.13^a^	2.32 ± 0.07
21-day body weight (kg)	3.19 ± 0.11^b^	3.66 ± 0.20^a^	3.35 ± 0.11
ADG (kg/d)	0.10 ± 0.00	0.12 ± 0.01	0.11 ± 0.00

Different lowercase letters indicate significant difference (*p* < 0.05) and capital letters indicate significant difference (*p* < 0.01).

### 3.3 The subcellular distribution of *NR4A1*

**Figure 3:**
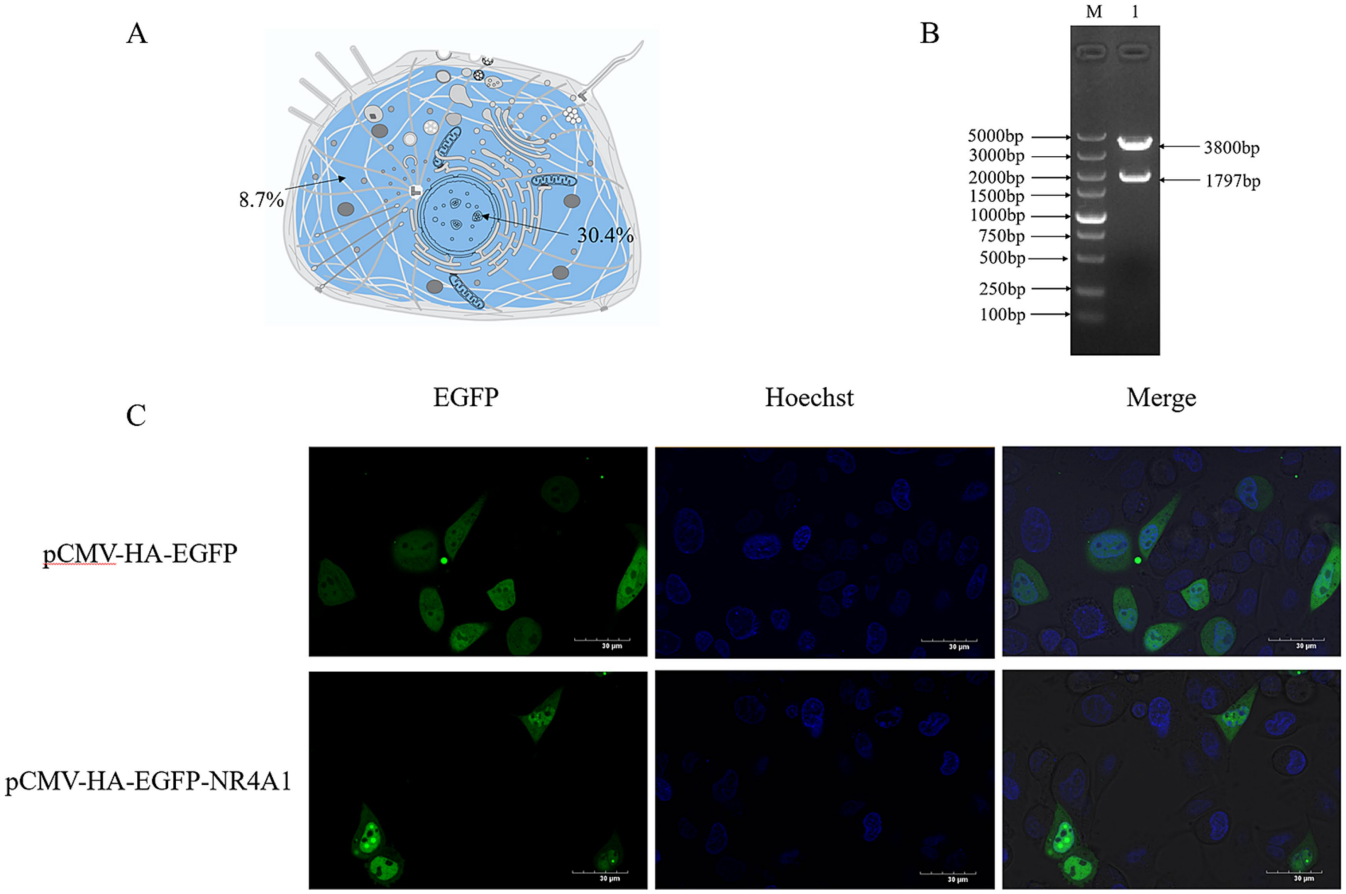
The subcellular distribution of *NR4A1*. **(A)** Prediction of cellular localization of *NR4A1*. **(B)** Construction of pCMV-HA-NR4A1. **(C)** The distribution of NR4A1-EGFP in the IPEC-J2 cell. Scale bar: 30 μm.

Based on UniProt, Figure 3A presents a schematic of the subcellular localization of NR4A1. Using PSORT II, the subcellular localization of *NR4A1* was predicted as follows: 30.4% nuclear, 21.7% cytoplasmic, 13.0% mitochondrial, 13.0% Golgi, 8.7% endoplasmic reticulum, 8.7% vesicles of the secretory system, and 4.3% peroxisomal (Figure 3A). To clarify the subcellular distribution of *NR4A1* in swine cells, the 1797 bp coding sequence of *NR4A1* was amplified, inserted into eukaryotic expression plasmid pCMV-HA-EGFP and verified using double-restriction enzyme digestion (Figure 3B). The pCMV-HA-EGFP-NR4A1 expression vector was transfected into IPEC-J2. Fluorescence and confocal analyses in IPEC-J2 showed that porcine NR4A1-EGFP fusion proteins located within the cell nucleus and cytoplasm (Figure 3C), which was consistent with the prediction of PSORT II.

### 3.4 *NR4A1* promotes the mRNA expression of *ANXA5*

**Figure 4:**
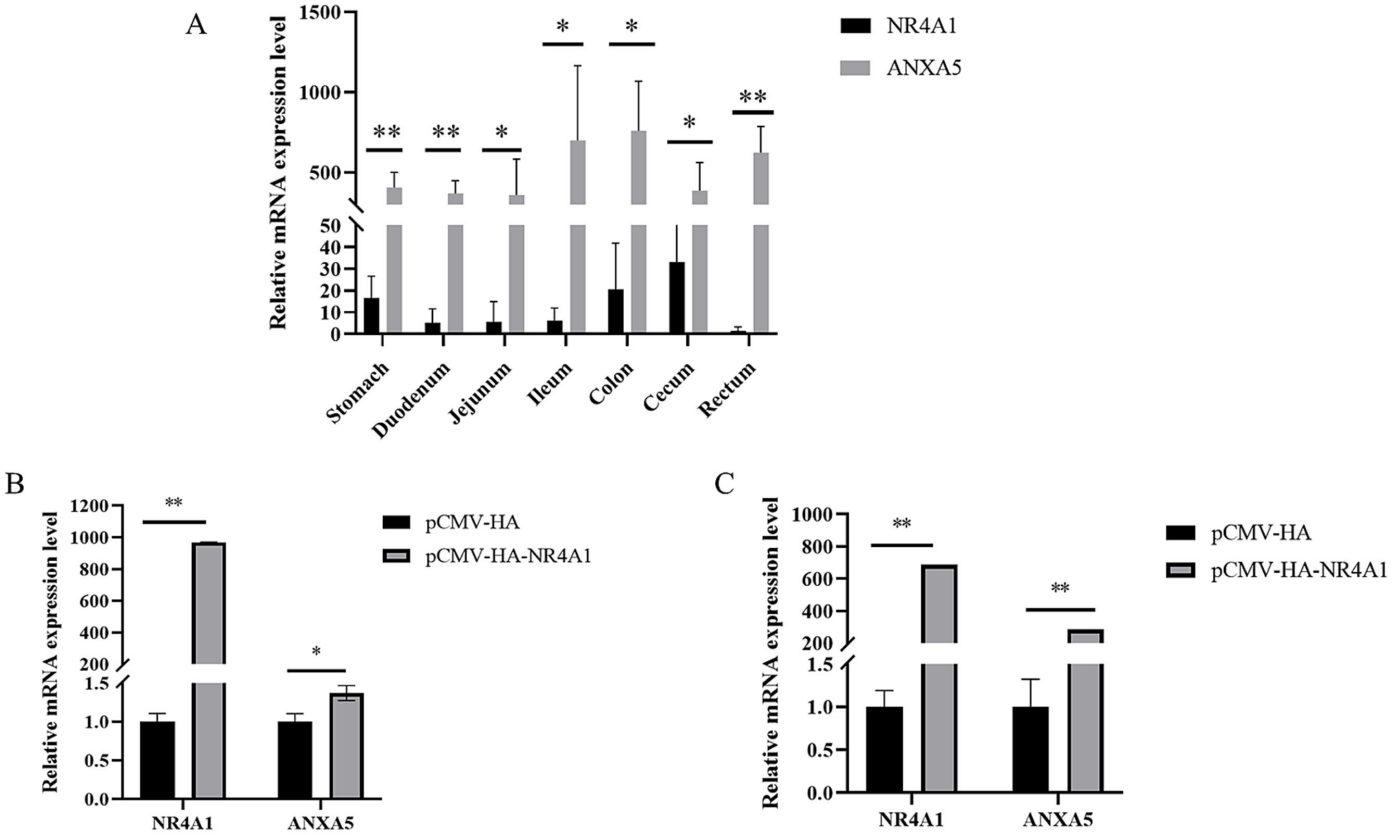
NR4A1 promotes the mRNA expression of *ANXA5*. **(A)** Tissue expression profiles of the porcine *ANXA5* and *NR4A1*. **(B)** Overexpression of *NR4A1* increases the mRNA expression of *ANXA5* in the IPEC-J2 cell. **(C)** Overexpression of *NR4A1* increases the mRNA level of *ANXA5* in the ST cell. Values were shown as the mean ± SEM (*n* = 3). *p < 0.05, **p < 0.01.

The mRNA expression of *NR4A1* and *ANXA5* in the gastrointestinal tract of weaned piglets was quantification by qRT-PCR. As shown in Figure 4A, both *NR4A1* and *ANXA5* were expressed in all the tissues including stomach, duodenum, jejunum, ileum, colon, cecum and rectum, where the expression of *ANXA5* were higher than that of the *NR4A1* gene (*p* < 0.05 or *p* < 0.01). Then, the *NR4A1* expression vector pCMV-HA-NR4A1 were constructed, verified and transfected into IPEC-J2 and ST. qRT-PCR showed that *NR4A1* overexpression significantly increased the mRNA expression of *ANXA5* in both IPEC-J2 (*p* < 0.01) and ST (*p* < 0.01) (Figures 4B,C).

### 3.5 Identification of the putative *NR4A1* binding site of porcine *ANXA5* promoter

**Figure 5:**
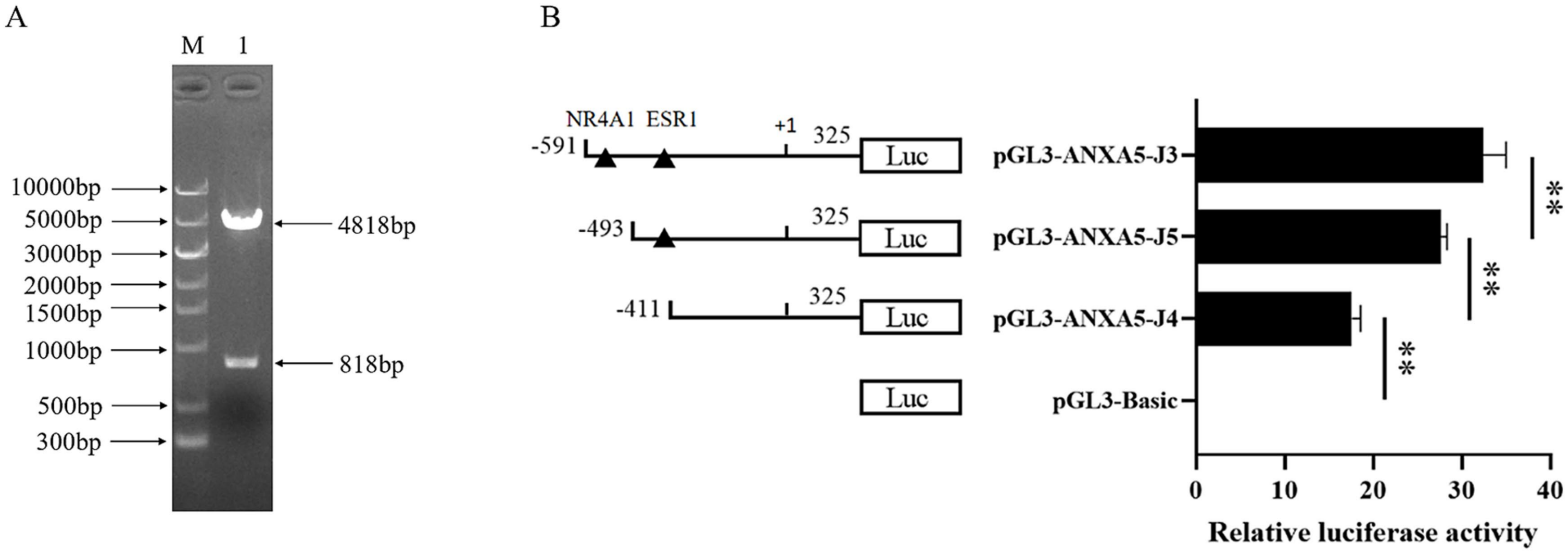
Identification of the putative NR4A1 binding site of porcine *ANXA5* promoter. **(A)** Construction of pGL3-ANXA5-J5. **(B)** Dual luciferase activity analysis of pGL3-ANXA5-J3 ~ J5. Values were shown as the mean ± SEM (*n* = 3). *p < 0.05, **p < 0.01.

To explore the function of this putative NR4A1 binding site, an 818-bp 5′-deletion fragment targeting the NR4A1 binding site was amplified to construct the corresponding pGL3-ANXA5-J5 (Figure 5A). Then, pGL3-ANXA5-J5, pGL3-ANXA5-J3 and pGL3-ANXA5-J4, were transfected into ST, respectively. As shown in Figure 5B, the luciferase activity of pGL3-ANXA5-J5 was weaker than that of pGL3-ANXA5-J3 (*p* < 0.01), but stronger when compared with pGL3-ANXA5-J4 (*p* < 0.01), indicating the promotive effect of the predictive NR4A1 binding region (−523 bp to -512 bp) on the transcription of porcine *ANXA5*.

### 3.6 g.-519 A > G modulates the *ANXA5* transcription partially through NR4A1

**Figure 6:**
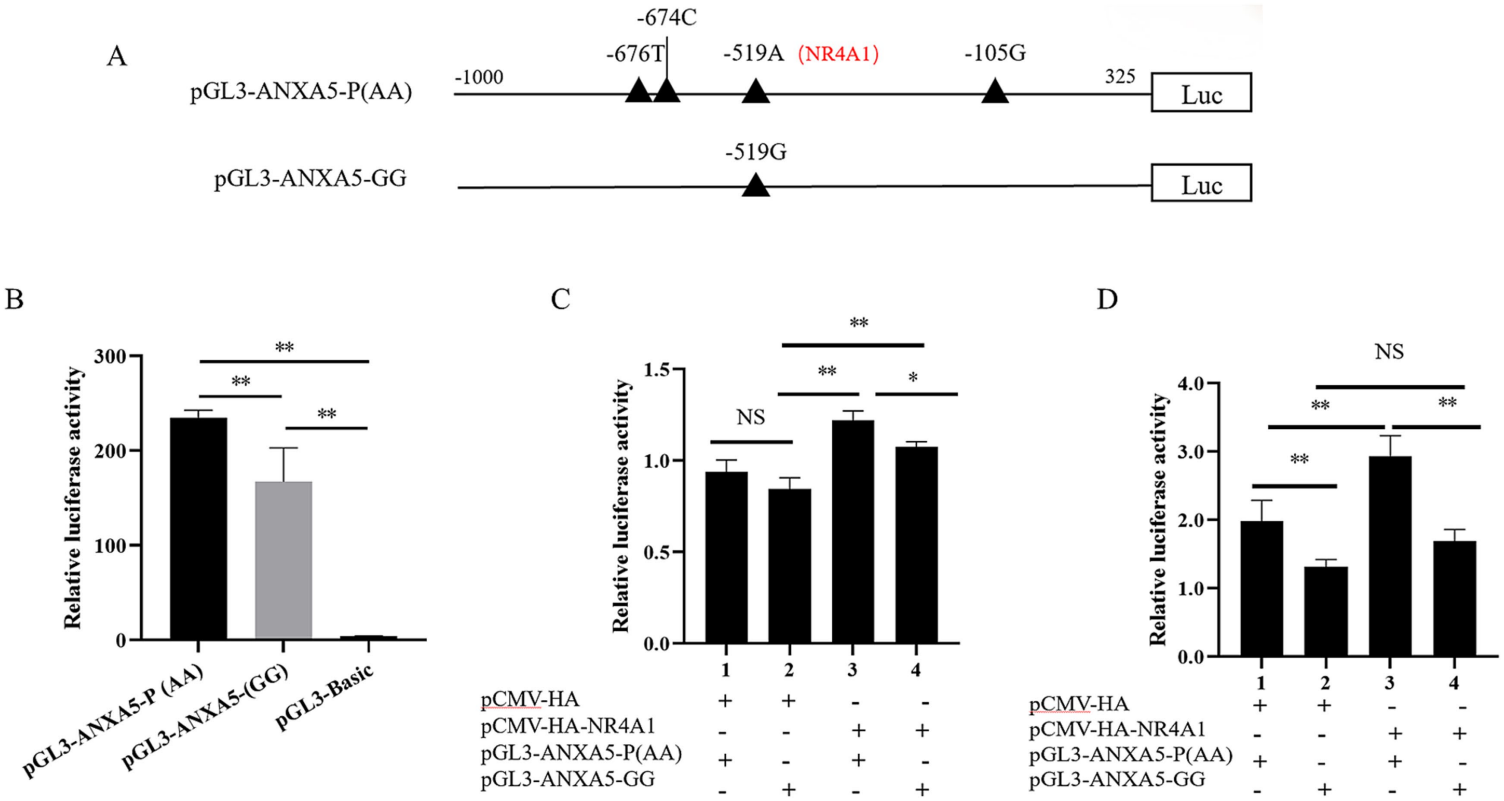
-519 A > G modulates the *ANXA5* transcription partially through NR4A1. **(A)** A schematic diagram of SNP point mutations in the promoter region of the porcine *ANXA5* gene. **(B)** Dual luciferase activity analysis of the SNP g.-519 A > G mutation plasmids. **(C)** Effect of *NR4A1* overexpression on the promoter activity of different alleles of SNP g.-519 A > G in IPEC-J2 cells. **(D)** Effect of the transcription factor *NR4A1* on the promoter activity of different alleles of SNP g.-519 A > G in ST cells. Values were shown as the mean ± SEM (*n* = 3). *p < 0.05, **p < 0.01.

According to our previous study, the luciferase reporter pGL3-ANXA5 containing A allele of SNP: g.-519 A > G, which was renamed as pGL3-ANXA5-P(AA) in the current study. To verify the effects of this SNP on *ANXA5* transcription, a fragment containing G allele was obtained based on pGL3-ANXA5-P(AA) and the mutation primer (Table 1) to construct pGL3-ANXA5-GG (Figure 6A). Both the pGL3-ANXA5-P(AA) and pGL3-ANXA5-GG were transfected into ST cells, respectively. Statistical analysis showed that the luciferase activity of pGL3-ANXA5-GG was lower than its wild type promoter (*p* < 0.01) (Figure 6B). Then, *NR4A1* expression vector was co-transfected into IPEC-J2 or ST with pGL3-ANXA5-P(AA) or pGL3-ANXA5-GG. As shown in Figure 6C, overexpression of *NR4A1* in IPEC-J2 significantly increased the luciferase activity of the pGL3-ANXA5-P(AA) (*p* < 0.01) or pGL3-ANXA5-GG (*p* < 0.01), and the luciferase activity of pGL3-ANXA5-P(AA) had higher luciferase activity when compared with pGL3-ANXA5-GG (*p* < 0.05). In the ST cells, *NR4A1* overexpression enhanced the luciferase activity of the pGL3-ANXA5-P(AA) (*p* < 0.01), but not the pGL3-ANXA5-GG (Figure 6D). Irrespective of *NR4A1* overexpression, the luciferase activity of pGL3-ANXA5-P(AA) remained higher than that of pGL3-ANXA5-GG (*p* < 0.01) in Figure 6D.

## 4 Discussion

### 4.1 Effects of g.-674 C > A and g.-519 A > G on piglet growth

Previous studies have revealed the effects of *ANXA5* polymorphism on reproduction traits in Yorkshires (9, 15), it is necessary to evaluate the genetic effect of *ANXA5* on piglets’ performance trait before the genome selection. In this study, a *Hha* I PCR-RFLP assay was established to detect an identified haplotype (SNP: g.-676 T > C, g.-674 C > A and g.-105G > T) by genotyping g.-674 C > A. According to Han et al. (15), the semen traits of AA animals were lower than AG boars. In the current study, although AA piglets had higher birth weight than the AC and CC animals, the g.-674 C > A was not associated with the later weight (from 3 day to the 21 day) and ADG. It seems the genetic selection based on g.-674 C > A might improve boar reproduction traits without negative effect on piglet weaning weight and growth. Additionally, this PCR-RFLP approach is more accurate and cost-effective when it compares with DNA sequencing. Hence, the *Hha*I PCR-RFLP is suggested to distinguish the identified haplotype in the promoter of *ANXA5*.

The abundance of genetic variation in local breeds is higher than that in commercial breeds. In current study, a novel g.-519 A > G in the promoter of *ANXA5* was identified in Min and Jinhua pigs, but fixed as A allele in Yorkshires and Durocs. It is known that the human-driven selection resulted in substantial phenotypic diversity among indigenous and commercial pigs (17–19). Hence, one possible explanation for the existence of g.-519 A > G in the Min pig and the Jinhua pig might be the weak artificial selection pressure. Chinese indigenous pigs are divided into North China, Central China, South Chinese, Lower Yangtze River Basin, Southwest, and Plateau, based on geographic distribution, historical origin and morphological characteristics (20, 21). The Min pig is well-adapted to the conditions of North China, while the Jinhua pig belongs to Central China. The geographic differences might be the second explanation for the allelic diversity among these breeds (17, 22, 23). Additionally, the heterozygous Min pig had better growth performance, which might be caused by the epistatic effect of non-alleles, or the interaction between the gene and environment. Min and Jinhua pigs’ high allelic diversity in *ANXA5* gene suggested the merit role of indigenous animals in swine genetic improvement.

### 4.2 g.-519 A > G regulates ANXA5 transcription partially by NR4A1

Nuclear receptor subfamily 4 group A Member 1 (NR4A1) is an immediate-early response gene which regulates diverse biological processes, including cell proliferation, apoptosis, and inflammatory responses (24). The current study demonstrated the existence of both *NR4A1* and *ANXA5* in the piglet’s intestine, and *NR4A1* promoted the transcription and expression of porcine *ANXA5* in IPEC-J2 cells. It is well known that the spatiotemporal expression of genes is closely linked to their functions. Hence, we presume that *NR4A1* might take important part in the intestine development or health partially by regulating the transcription of *ANXA5*, thereby affecting piglets’ growth. This hypothesis was supported by literature. For example, both *NR4A1* and ANXA5 were associated with ulcerative colitis (UC) (8, 25); *NR4A1* expressed in intestine tissues, inflammatory cells and epithelium (26, 27), regulated the differentiation and function of Paneth cell (28); *ANXA5* had differential expression in the jejunal mucosa of IUGR and normal piglets (10).

The subcellular localization of one protein partially determines its function. NR4A1 generally resides in the nucleus, but can be translocated to the mitochondria in specific cells or upon stimulation. For example, H_2_O_2_ (29), Celastrol (24) or Gly-Pro-Ala (GPA) peptide (30) can promote the nucleocytoplasmic shuttling of NR4A1, induces autophagy, apoptosis, inflammation or other biological process. Here, the NR4A1-EGFP fusion proteins were observed in the nucleus and cytoplasm, indicating that *NR4A1* might regulate ANXA5 as a transcription factor in IPEC-J2 cells.

Nowadays, it has been accepted that non-coding regions harbor abundant variations (31–33). For example, g.128G > A influenced granulosa cell apoptosis and sow fertility by altering the RBP-Binding sit of LncRNA NORSF (34); g.-283G > C and g.-271C > T in the miR-23a promoter affected Large White fertility traits partially by altering the transcription of miR-23a in porcine GCs (35). The roles of promoters and enhancers in complex traits have emphasized by accumulating swine genome-wide chromatin landscapes (36–38). The current study focused on the cis-elements of porcine *ANXA5*, the increased effect of *NR4A1* on the luciferase activity of the A allele suggested that g.-519 A > G might alter *ANXA5* expression dynamics partially through the modulation of NR4A1 binding. It should be noted that A allele has high transcription activity, but the heterozygote piglets exhibit higher weight. The explanation is as follows: Firstly, post-transcriptional regulation or post-translational modifications of ANXA5 potentially weaken the functional impact of g.-519 A > G; Secondly, allelic interactions (e.g., overdominance effect) strengthen heterozygote phenotypes. Additionally, although g.-519 A > G was identified as a functional variant, it is still unclear whether this SNP alters the transcription of *ANXA5* by binding the cis-element or interacting with other transcriptional factors or small RNA. Further study will focus on clarifying the regulatory mechanism of *ANXA5*. Given the pleiotropic nature of *ANXA5*, future studies are warranted to evaluate the genetic effects or function of g.-519 A > G on Min pigs’ reproductive trait.

## 5 Conclusion

This study demonstrated that both g.-674 C > A and g.-519 A > G were associated with piglets’ weight in the Min pig, and it is efficient and convenient to genotype g.-674 C > A by *Hha* I PCR-RFLP. Porcine NR4A1 was located in the nucleus and cytoplasm, regulated the transcription and expression of *ANXA5* in piglet intestinal epithelial cells. SNP: g.-519 A > G might have an allele-specific effect on *ANXA5* transcription partially by varying affinity for NR4A1.

## Data Availability

The datasets presented in this study can be found in online repositories. The names of the repository/repositories and accession number(s) can be found in the article/supplementary material.

## Footnotes

^1^ http://afs.okstate.edu/breeds/swine/minzhu/index.html/

^2^ https://www.lynnon.com/

^3^ https://jaspar.elixir.no/

^4^ https://www.genscript.com/psort.html

^5^ https://www.uniprot.org/
